# Cochlear resident macrophage mediates development of ribbon synapses *via* CX3CR1/CX3CL1 axis

**DOI:** 10.3389/fnmol.2022.1031278

**Published:** 2022-11-28

**Authors:** Xinyu Song, Yang Li, Rui Guo, Qianru Yu, Shan Liu, Qi Teng, Zhong-Rui Chen, Jing Xie, Shusheng Gong, Ke Liu

**Affiliations:** ^1^Department of Otolaryngology Head and Neck Surgery, Beijing Friendship Hospital, Capital Medical University, Beijing, China; ^2^Clinical Center for Hearing Loss, Capital Medical University, Beijing, China

**Keywords:** cochlear macrophage, ribbon synapses, CX3CR1/CX3CL1, development, hearing Loss

## Abstract

Cochlear ribbon synapses formed between spiral ganglion neurons and inner hair cells in postnatal mice must undergo significant morphological and functional development to reach auditory maturation. However, the mechanisms underlying cochlear ribbon synapse remodeling remain unclear. This study found that cochlear resident macrophages are essential for cochlear ribbon synapse development and maturation in mice *via* the CX3CR1/CX3CL1 axis. CX3CR1 expression (a macrophage surface-specific receptor) and macrophage count in the cochlea were significantly increased from postnatal day 7 then decreased from days 14 to 28. Seven-day treatment with CX3CR1 inhibitors and artificial upregulation of CX3CL1 levels in the inner ear environment using the semicircular canal injection technique were initiated on day 7, and this resulted in a significant increase in hearing threshold on day 28. Additionally, abnormalities in the morphology and number of cochlear ribbon synapses were detected on day P14, which may be associated with hearing impairment. In conclusion, macrophage regulation of cochlear ribbon synapse remodeling *via* the CX3CR1/CX3CL1 axis is required during hearing development and offers a new perspective on immune-related hearing loss throughout auditory development. Importantly, it could be a new treatment target for sensorineural hearing loss.

## Introduction

The ribbon synapse of the inner hair cells is the first excitatory afferent synapse in the auditory pathway and is critical for sound encoding and transmission from the cochlea to the brain (Lu et al., 2016; Coate et al., 2019). The number of cochlear ribbon synapses continuously decreases after peaking at P7-P10. This process, known as synaptic pruning, involves axonal fine-tuning and contraction of immature spiral ganglion neurons terminals (Yu et al., 2021). We previously found that autophagy may be necessary for the remodeling of ribbon synapses in cochlear inner hair cells (IHCs) before the onset of hearing. Autophagy serves as one of the primary mechanisms of hearing development and maturation in the postnatal cochlea. In this study, autophagy in IHCs was regulated by rapamycin and 3-methyladenine, autophagy activators and inhibitors, respectively. We found that normal hearing function could not be established in treated mice, coupled with immature ribbon synapses in cochlear IHC (Xiong et al., 2020). However, the mechanism is yet to be fully elucidated owing to the complexity of the molecular mechanism of ribbon synapse pruning and maturation.

Current research indicates that microglia, the resident macrophages of the brain, play a direct role in synaptic pruning in the central nervous system (CNS) (Kempf and Schwab, 2013; Brites and Fernandes, 2015; Kim et al., 2017). The role of microglia in the CNS has been extensively studied. In the adult brain, microglia interact with the neurons and synapses. (Favuzzi et al., 2021; Basilico et al., 2022) Microglia engulf and eliminate dying cells as part of their well-known phagocytosis, but it has also been demonstrated that they eliminate weakly active synapses. Several chemokine signaling pathways, especially the fractalkine (CX3CL1) signaling system, regulate these interactions (Korbecki et al., 2020; Pawelec et al., 2020). CX3CR1 is a leukocyte surface protein that is most abundant in monocytes and macrophages in various organs (Leonardi et al., 2018). CX3CL1 is secreted mainly by endothelial cells and neurons, and CX3CR1 is its specific receptor (Cormican and Griffin, 2021).

Macrophages and mononuclear phagocytes are major players in the cochlear innate immune system and are responsible for mediating pathogen detection and elimination, tissue homeostasis, and injury response (Hu et al., 2018). The immune defense function of cochlear macrophages in the inflammatory environment following various pathological injuries have been extensively studied. These injuries include auditory damage, ototoxicity, immune attack, and mechanical damage caused by cochlear implantation, which all cause an inflammatory response in the cochlea. The development of new genetic and functional analysis tools has revealed the presence of cochlear-resident immune cells. It was found that approximately 62% of cochlear resident macrophages are located in the neural tissue and approximately 36% are located at the edges of the spiral ligament and bone spiral plate. Macrophages are evenly distributed along a gradient from the apex to the base of the cochlea, with few aggregates or gaps in macrophage distribution. This spatial configuration ensures that each cell has an immune examination area and that all cochlear regions are under immune surveillance (Okano et al., 2008). However, the function of cochlea resident macrophages has not yet been clarified.

This study hypothesized that macrophages are involved in synaptic pruning *via* the CX3CL1-CX3CR1 axis. Phagocytosis of macrophages during synaptogenesis may be necessary for IHC synaptic remodeling in the cochlea before the onset of hearing. We found that the increasing and decreasing trends in the number of macrophages residing in the cochlea matched with the trends in synaptic pruning, with macrophages residing in the cochlea mainly clustered near the auditory nerve and ribbon synapses during the peak abundance of synapses. To control macrophage pruning in cochlear synapses, CX3CR1 receptor inhibitors were injected, and CX3CL1 levels were artificially increased in the inner ear environment. CX3CR1 inhibitor treatment increased the number of synapses remaining after cochlear synaptic pruning. In contrast, CX3CL1 upregulation reduced the number of synapses in the cochlea. Neither treatment resulted in normal hearing.

## Materials and method

### Animals

C57BL/6J pregnant mice were obtained from Beijing Vital River Laboratory Animal Technology Co. Ltd. 87 postnatal mice were included in this experiment. All animal experiments were approved by the Animal Ethics Committee of Capital Medical University and were performed strictly by the standards of the Animal Ethics Committee.

### Drug administration

AZD8797 (MCE, KAND567), an inhibitor of CX3CR1, was dissolved in DMSO as a stock solution (10 mM/ml) and stored at-20°C. Before use, AZD8797 was diluted with Corn oil. Animals in each group received intraperitoneal injections of AZD8797 at a dose of 1 mM/kg daily from P7 to P14 consecutively, the controls were administered with the same amount of DMSO with Corn oil.CX3CL1 (peprotech, 300-31) was dissolved in pure water as a stock solution (1 mg/ml) and stored at −20°C. For P7 mice, 0.6ul of concentrated storage solution was diluted with water to 2 μl of working solution before injecting the drug into the semicircular canal.

### Auditory brainstem response

Intraperitoneal injection of ketamine and xylazine (100 mg/kg of ketamine and 10 mg/kg of xylazine) was used to anesthetize the animals. A warming blanket was used to keep the body temperature at 37.5°C. Subdermally applied stainless steel needle electrodes were positioned posterior to the animal’s stimulated and unstimulated ears (inverting input and ground) and above the vertex (noninverting input). The amplitude was detected at 90 dB SPL for each stimulus frequency during an ABR test using System 3 hardware and SigGen/BioSig software (TDT, United States). The threshold was determined by the lowest stimulus intensity that produced a repeatable ABR wave.

### Immunofluorescence

The cochleae were extracted and fixed with 4% paraformaldehyde in phosphate-buffered saline (PBS) for 1 h before the audiological examinations. After that, the basilar membranes were meticulously cut apart. Samples were cleaned with PBS before being incubated at room temperature with 0.3% TritonX-100 for 30 min and 10% goat serum (ZSGB-BIO) for 1 h. The preparation were incubated overnight at 4°Cwith primary antibodies including: rat anti-F480 (1:50, Abcam, MAB386), rabbit anti-CX3CR1 (1:50, Santa, sc-377227),mouse anti-GluA2 (1:400, Millipore, MAB397), mouse anti-CtBP2 (1:500, Abcam, ab204663). The next day, samples were carefully washed three times with PBS for 10 min each and incubated with Alexa FluorTM 488, 568, or 647 (1:300) conjugated secondary antibodies for 2 h at room temperature, followed by three washes with PBS for 10 min each and mounted on a glass coverslip with DAPI (ZSGB-BIO, ZLI-9557).

### Laser confocal microscopy

Images were scanned with a × 60 oil-immersion confocal microscope (TCS SP8 II; Leica, Wetzlar, Germany). All Scanning was performed from top to bottom with an interval of 0.5 μm/layer, and a series of z-stack images were acquired.

### Quantification of the immunofluorescence signals

In order to calculate the average number of ribbon synapses for each IHC, the total numbers of GluA2- and CtBP2-stained puncta were added together and divided by the total number of IHC nuclei.

### Quantitative analysis of macrophage morphology and distribution

The size and morphology of macrophages are determined by distinctive F4-80 expressions. Greater in size than other types of leukocytes and having uncommon shapes like dendritic, amoeboid-curved, or aberrant morphologies with protrusions. We employed a well-known specialized surface stain called F4-80 to positively stain the critical cells in order to determine their distribution. A subset of cochlear macrophages includes macrophages in the neural tissue, basement membrane macrophages (BM-macrophages), and extravascular lateral wall macrophages. Using bright-field illumination and a fluorescence microscope, each macrophage was distinguished as a subset of the cochlear macrophage population. The osseous spiral layer, the basal membrane, and the lateral wall of the vasculature can all be distinguished visually as parts of the cochlea. By counting the number of cells present in a sample of 0.1 mm^2^ in each of the three anatomical cochlear turns (apical, middle, and basal) per specimen, distribution analyses for macrophages at neural tissue, basement membrane macrophages (BM-macrophages), and extravascular lateral wall were carried out. After computing the mean for these counts, an average value per unit area in each cochlea was generated. Cell counts per unit area across specimens for each group were averaged to obtain group means.

### Western blot analysis

Hank’s balanced salt solution was used to newly dissect the cochlea after it was quickly removed (Gibco, 1491037). The cochlea was homogenized in ice-cold RIPA lysis solution (G2002, Servicebio) with phosphatase inhibitor cocktails and RIPA lysis buffer base (G2007, Servicebio), After 30 min on ice, tissue fragments were separated by centrifugation at 12,000 *g* for 10 min at 4°C, with the supernatants being kept as the total protein fractions. The BCA Protein Assay Kit was used to measure the protein concentrations (G2026, Servicebio). For every sample, two cochleas from the same mouse were combined. Primary antibody concentrations were anti-CX3CR1 (1:500; Abcam, ab8020) and anti-GAPDH (1:1000; AF5009, Beyotime). Protein samples (10 ll) were separated using SDS-PAGE (G2003, Servicebio). The proteins were then transferred onto nitrocellulose after electrophoresis. The membrane that was blocked using a 5% nonfat dry milk solution in 0.5% TBST. The membranes were exposed to primary antibodies against CX3CR1 (1:500) or anti-GAPDH (1:1000) for overnight incubation at 4°C, followed by three TBST washes lasting 10 min each. Secondary antibodies were applied to the membranes and incubated for 1 h at a concentration of 1:6000. The membrane was thoroughly washed before the immunoreactive bands could be seen using ECL. Using AlphaEase FC software, X-ray films of Western blots were scanned and examined. Using the optical density ratio of the CX3CR1 band within the GAPDH bands, the relative expression rates of several samples were examined.

## Result

### Inner hair cells undergo synaptic pruning before auditory maturation

**Figure 1:**
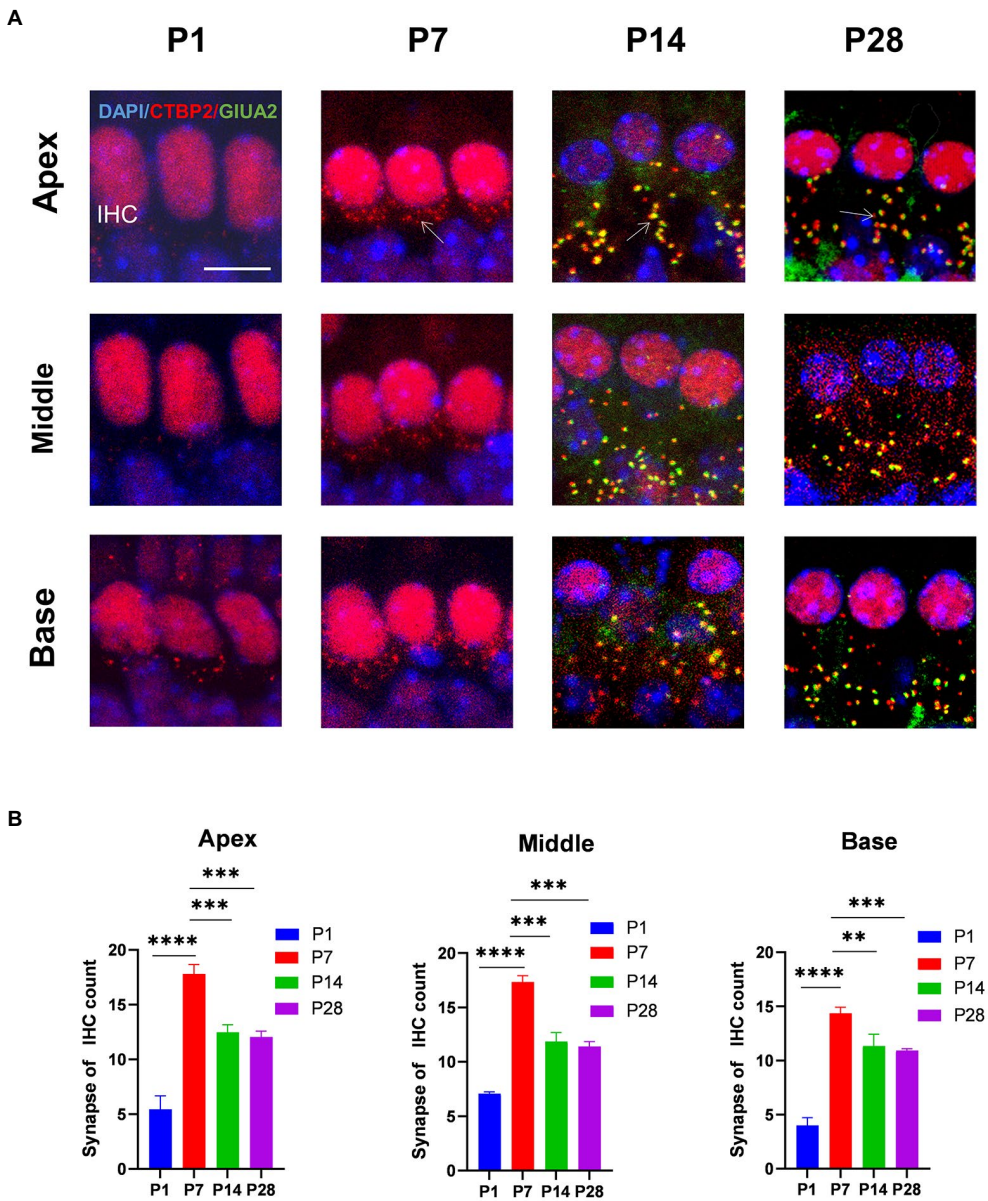
The number of synapses during postnatal mouse cochlear development is detected from P1 to P28. **(A)**: ctbp2 dots (red) and glua2 dots (green) on immunofluorescence staining show the dynamic changes in presynaptic and postsynaptic proteins on P1, P7, P14, and p28. Scale bar = 5 μm, *n* = 3. The image at the bottom is an enlarged image of macrophages. **(B)** The number of synapses per IHCs is increased from birth to P7 until P14, after which the number of synapses are stabilized. **p < 0.01, ***p < 0.001, ****p < 0.0001.

To determine the trends in the number of cochlear ribbons during synaptic pruning, presynaptic proteins were labeled with an anti-RIBEYE/CtBP2 antibody and postsynaptic proteins with an anti-GluA2 antibody. In the initial state (p1-p7) of ribbon synapse formation in the cochlea, the presynaptic dots were in the vicinity of the subnuclear or perinuclear region then gradually decreased to the basal level of the IHC cytoplasm at P14. Fewer presynaptic ribbons appeared at P1, and a higher number of presynaptic ribbons appeared around P7. The number of presynaptic ribbons gradually decreased from P14, after which the number remained stable (Figures 1A,B). Additionally, postsynaptic proteins were expressed at P14.

### Synaptic pruning is accompanied by a high degree of macrophage activation

**Figure 2:**
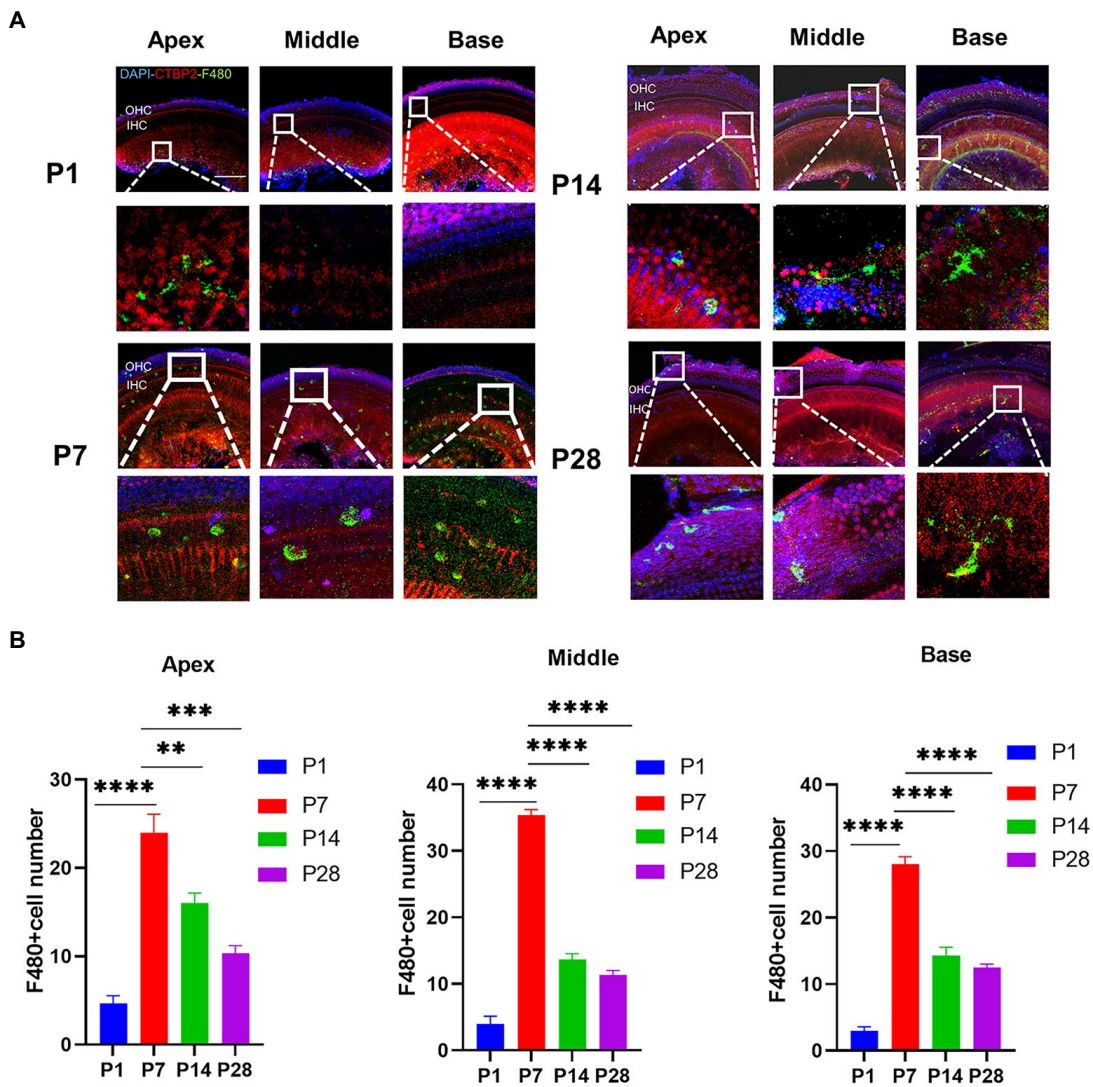
The number of macrophages during cochlear development in postnatal mice is detected from P1 to P28. **(A)** Immunofluorescence staining (green) is used to detect the dynamic changes in the number of macrophages on P1, P7, P14, and p28. Scale bar = 50 μ m, *n* = 3. At P7, macrophages have a round shape, while they have a dendritic shape after P14. **(B)** The number of macrophages is increased from birth to P7 and then decreased until P14. **p < 0.01, ***p < 0.001, ****p < 0.0001.

To confirm the timing of the presence of cochlear resident macrophages, they were labeled with an anti-F4-80 antibody. The number of cochlear macrophages peaked at P7, were distributed near the cochlear basilar membrane and auditory nerve, and were clustered near the cochlear ribbon synapses of the inner hair cells. Macrophages were usually circular and amoeboid in shape at the newborn stage, which was associated with phagocytosis. As the cochlear ribbon synapse and auditory nerve developed and matured, the number of macrophages decreased from P14, and the distribution migrated from the basilar membrane to the lateral wall of the blood vessels and auditory nerve. The number of macrophages stabilized within a certain range (Figures 2A,B).

### CX3CR1 plays a key role in the activation and recruitment of macrophages during the development

**Figure 3:**
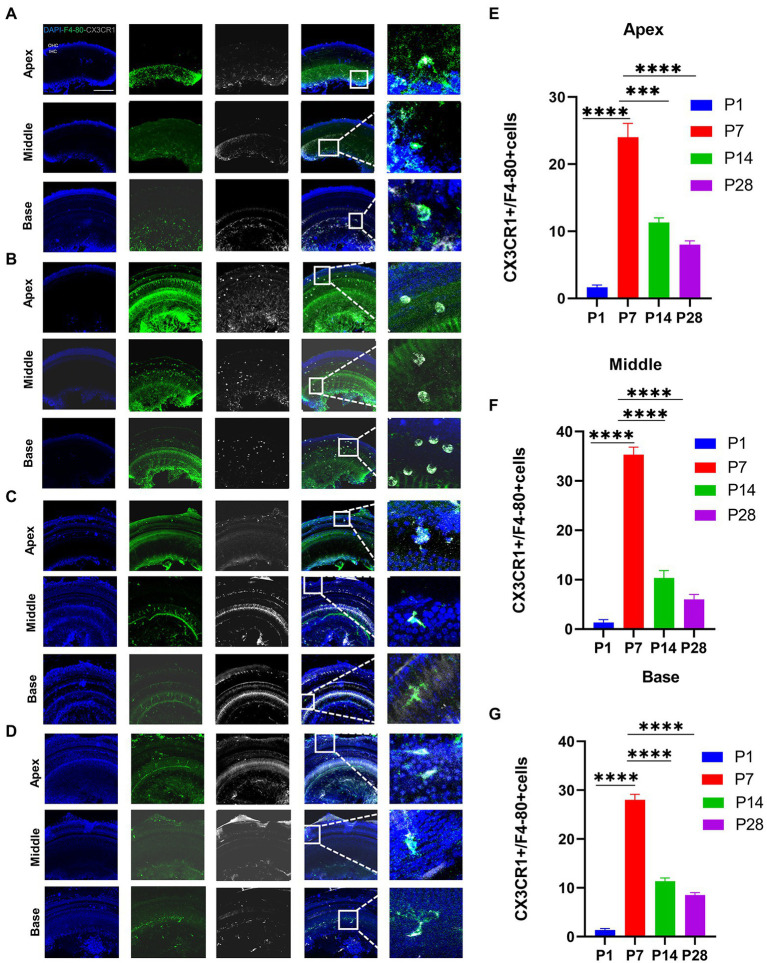
Detection of the number of CX3CR1 + macrophages during cochlear development from P1 to P28 in postnatal mice. **(A)** Immunofluorescence staining is used to detect the number of CX3CR1 + macrophages and identify the dynamic changes in the number of macrophages in P1. CX3CR1 (grey) colocalized with f4-80 (green). The nuclei are stained with DAPI. **(B)** Immunofluorescence staining is used to detect the number of CX3CR1 + macrophages and identify the dynamic changes in the number of macrophages at P7. **(C)** Immunofluorescence staining is used to detect the number of CX3CR1 + macrophages and identify the dynamic changes in the number of macrophages at P14. **(D)** Immunofluorescence staining is used to detect the number of CX3CR1 + macrophages and identify the dynamic changes in the number of macrophages at P28. Scale bar = 50 μm, *n* = 3. The image on the right is an enlarged image on the left. **(E–G)** Quantitative analysis of the number of CX3CR1 + macrophages from P1 to P28 showing that the number of CX3CR1 + macrophages from the apical to the middle to the basal turn of the cochlea is significantly higher on P7 than on P1, P14, and P28. **p < 0.01, ***p < 0.001, ****p < 0.0001.

**Figure 4:**
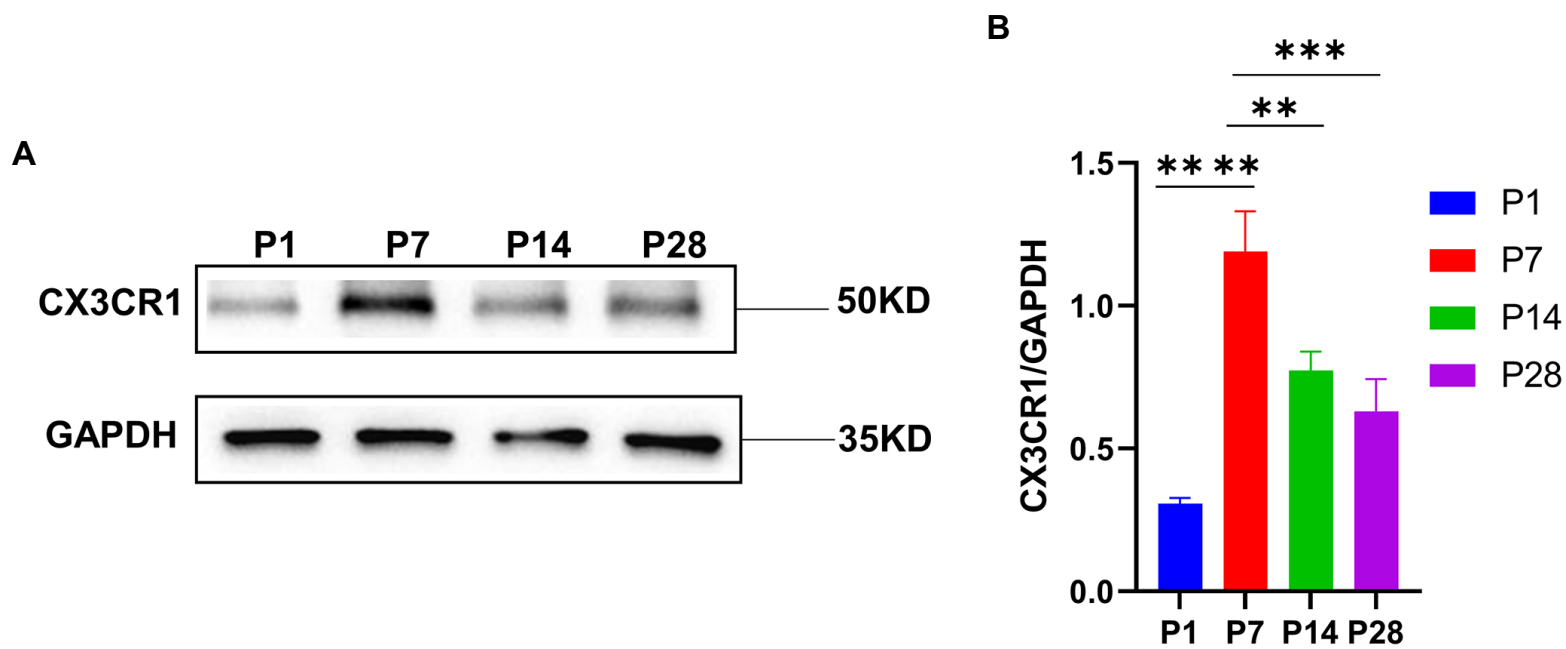
**(A)** CX3CR1 expression in the cochlea of postnatal mice is determined by western blotting. A significantly stronger band is identified around P7 than in P1, P14, and P28 (*n* = 3). **(B)** Quantitative analysis of CX3CR1 expression from P1 to P28 showing that the ratio of CX3CR1 to GAPDH is significantly higher in P7 than in P1, P14, and P28. ****p < 0.0001,**p = 0.0019, ***p = 0.0003. GAPDH is used as the loading control (*n* = 3).

To investigate the key role of CX3CR1 in cochlear macrophage activation, migration, and phagocytosis, cochlear macrophages were labeled with anti-CX3CR1 and anti-F4-80 antibodies. The results showed that macrophages almost completely expressed CX3CR1 receptors at P7. Quantification of the double-positive cells showed that the number of CX3CR1+ and F4-80+ double-positive cells was markedly higher at P7 than that at other periods before hearing maturation (Figures 3A–G). This indicated that the trend of CX3CR1-positive macrophages was consistent with the trend of the number of synapses. Correspondingly, when whole cochlear tissue was used for quantitative western blotting analysis, CX3CR1 expression was also observed to be much higher at P7 than that at other periods before hearing development (Figures 4A,B). This suggested that CX3CR1 was highly expressed in activated and migratory active macrophages and was involved in the critical process of synaptic pruning.

### Absence of CX3CR1 blocks synaptic pruning and increases the number of synapses

**Figure 5:**
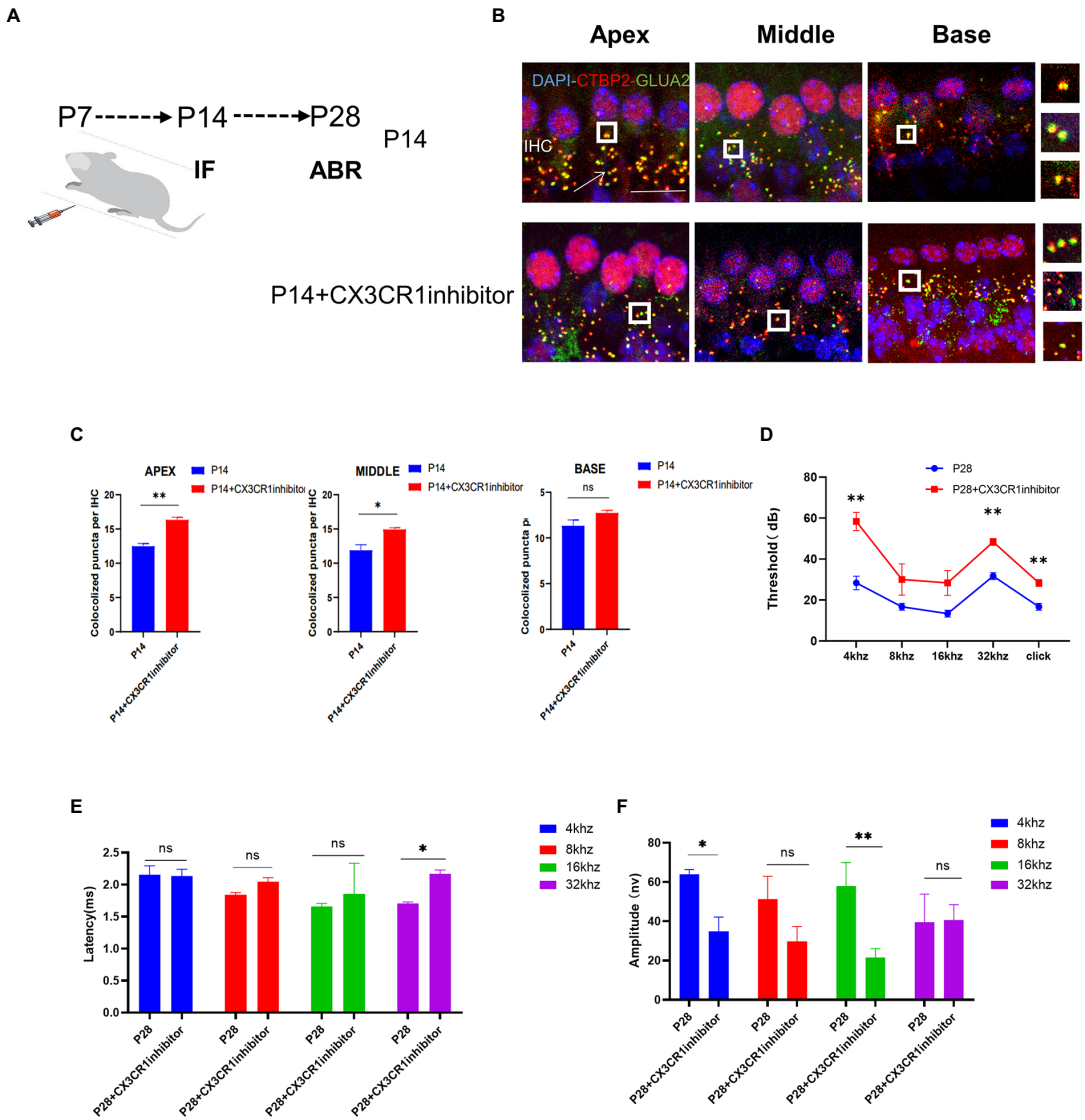
Inhibition of CX3CR1 activation in postnatal mice significantly impairs the morphological features of the ribbon synapses in cochlear IHC. **(A)** Mice were intraperitoneally injected with CX3CR1 inhibitor or normal saline daily starting from 7 days after birth. **(B)** Immunofluorescence staining of IHC ribbon synapses at P14 in each group. Presynaptic and postsynaptic proteins are co-stained; the nucleus is stained with DAPI. A magnified view of the synaptic sites is shown on the right. **(C)** Quantitative analysis showing that the number of synapses in the apex to the middle and bottom synapses in the cochlea is significantly higher in the CX3CR1 inhibitor-treated group than in the control group (*p < 0.05, **p < 0.01). Scale bar = 10 μm, *n* = 3. **(D–F)** Quantification of ABR threshold, amplitude, and latency of ABR wave I on P14 in control mice and CX3CR1 inhibitor mice. Significant increases in ABR thresholds at 4 and 32 kHz and click frequencies are detected in treated mice and controls. The latency of ABR wave I across frequencies (32 kHz) at P14 is longer in the treated mice than in controls. The amplitude of ABR wave I across frequencies (4 and 16 kHz) at P14 is lower in treated mice than in control mice (treatment group: *n* = 4, control group: *n* = 4; *p < 0.05, **p < 0.01).

To determine whether CX3CR1 receptors play a key role in synaptic pruning before the maturation of cochlear IHC hearing function. The CX3CR1 inhibitor was injected continuously for 7 days since P7 (Figure 5A). At P14, mice injected with the CX3CR1 inhibitor showed decreased expression of CX3CR1 protein (Supplementary Figure 1). In addition, the treatment group showed a significant increase in pre-synaptic and post-synaptic signals in the apical, middle, and basal turns (Figure 5B). These results showed that in resident macrophages in the cochlea, injection of the CX3CR1 receptor inhibitor led to a large amount of synaptic signal accumulation (Figure 5C). These findings showed that ribbon synaptic pruning and remodeling of cochlear IHC were impaired after deletion of CX3CR1 expression. Next, we investigated whether the inhibition of CX3CR1 expression could significantly affect the development and maturation of hearing. Auditory brainstem response (ABR) tests were performed in treated mice and control mice at 28 days postnatally. It was found that the ABR thresholds were significantly higher, the ABR I-wave amplitude was decreased, and the ABR I-wave latency period was extended in the treatment group (Figures 5D–F). This suggested that the inhibition of CX3CR1 expression before hearing onset may severely affect the development of hearing and lead to hearing loss.

### CX3CL1 upregulation promotes synaptic pruning

**Figure 6:**
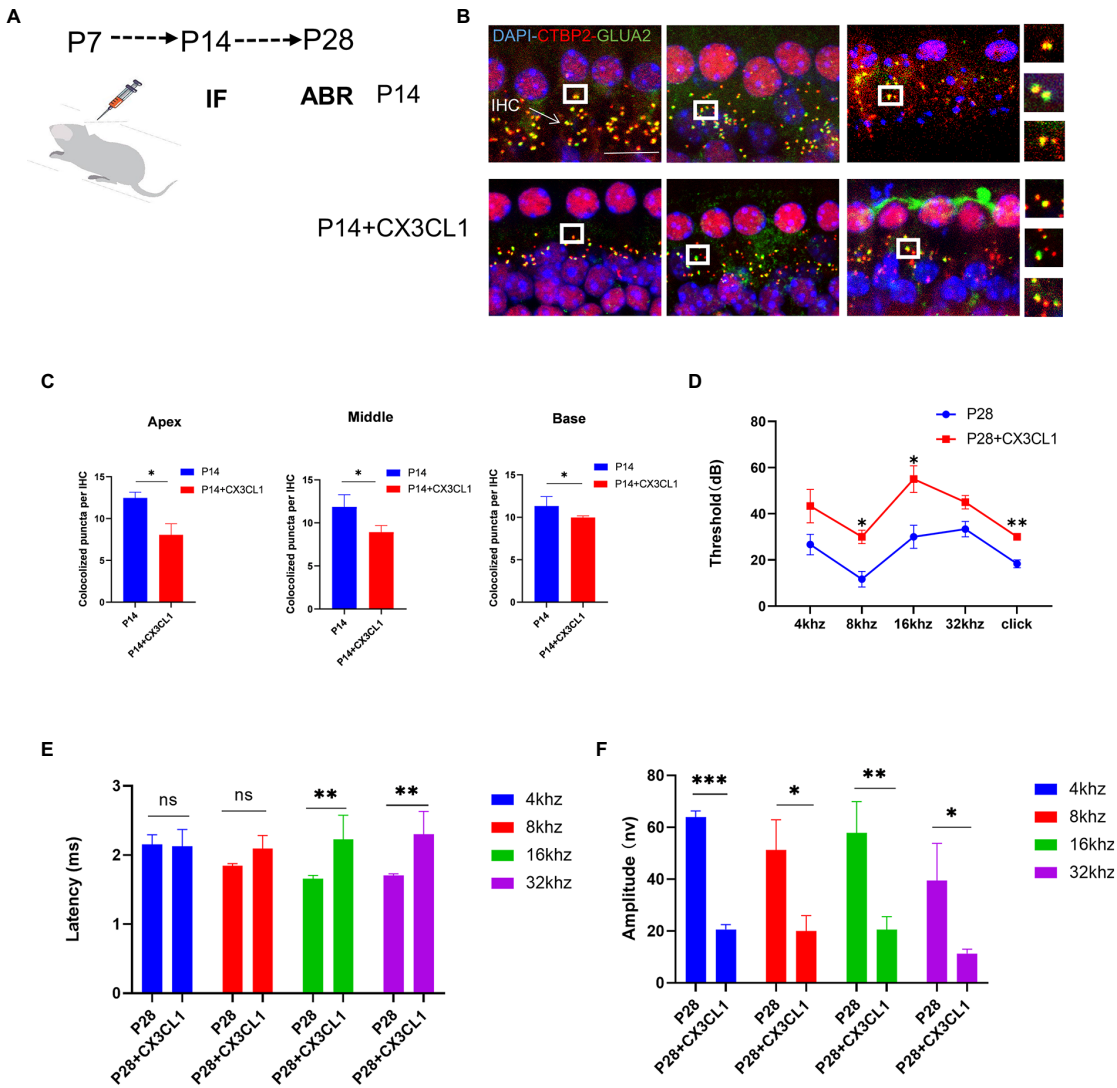
CX3CL1 overexpression in postnatal mice significantly impairs the morphological features of ribbon synapses in cochlear IHC. **(A)** CX3CL1 is injected into the semicircular canal of mice 7 days after birth **(B)**. IHC ribbon synapses in each group are subjected to immunofluorescence staining at P14. Presynaptic protein and postsynaptic protein are co-stained; the nucleus is stained with DAPI. The right side is the enlarged image of the synaptic point. **(C)** Quantitative analysis of synapse numbers showing that the number of synapses in the apex to the middle and bottom synapses in the cochlea is significantly higher in the CX3CL1 treatment group than in the control group (*p < 0.05, **p < 0.01). Scale bar = 10 μm, *n* = 3. **(D–F)** Quantification of ABR threshold, amplitude, and latency of ABR wave I in control mice and CX3CL1 mice on P14. Significant increases in ABR thresholds at 8 and 16 kHz and click frequencies are detected in both treated mice and control mice. The latency of ABR wave I across frequencies (16 and 32 kHz) at P14 is longer in treated mice than in control mice. The amplitude of ABR wave I across frequencies (4, 8, 16, and 32 kHz) at P14 is lower in treated mice than in control mice (treatment group: *n* = 4, control group: *n* = 4; *p < 0.05, **p < 0.01).

We investigated whether CX3CL1 upregulation in mice at 7 days postnatal interferes with the synaptic pruning function of cochlear resident macrophages. CX3CL1 is secreted by neurons and has an association with spiral neurons and macrophages. Macrophages were labeled in frozen sections, and on P28, macrophages were mainly distributed near the spiral ganglion and the lateral wall of the blood vessels in the cochlear sections of mice (Supplementary Figure 2). Thus, with respect to spatial effects, CX3CL1 secreted by spiral neurons is more likely to bind to receptors on the surface of cochlear-resident macrophages. We exogenously administered CX3CL1 to P7 mice by posterior semicircular canal surgery (Figure 6A). At P14, the number of sub-IHC band synapses was significantly lower in CX3CL1-treated mice than in control mice (Figures 6B,C). There was also no difference in the number of cochlear synapses at P14 between the treatment and control groups when pure water was introduced through the semicircular canal (Supplementary Figure 3). Overall, our results suggest that CX3CL1 upregulation enhanced synaptic pruning, resulting in a significant reduction in the number of synapses.

Next, it was investigated whether the exogenous administration of CX3CL1 could significantly affect the development and maturation of hearing. At P28, the ABR threshold was significantly higher, the ABR I-wave amplitude was decreased, and the ABR I-wave latency period was extended in the treatment group (Figures 6D–F). This suggested that CX3CL1 upregulation before hearing onset may severely affect hearing development and maturation, leading to hearing loss. The first week after birth is a critical period for auditory development in mice, and the most important feature of this period is synaptic remodeling, which occurs as synaptic pruning and refinement of redundant synapses, as demonstrated in our study. Within a month of birth, the modification of cochlear ribbon synapses is completed. In adult mice, synaptic pruning activity is reduced or absent, as shown by the maintenance of synaptic quantity at a steady level, which denotes the completion of auditory development.

## Discussion

Wong et al. reported that in the initial state of ribbon synapse formation in the cochlea, the presynaptic dots around the subnuclear or perinuclear region are also replaced by a “floating” ribbon. By the second week of life, these presynaptic dots gradually descend to the basal level of the IHC cytoplasm, providing a plausible explanation for the majority of synaptic signals in the basal region of the IHC. Furthermore, the number of presynaptic signals peak 1 week postnatally and then decrease until they stabilize (Wong et al., 2014). Our study also confirmed the changes in cochlear ribbon synapse morphology and number before hearing maturation. Macrophages are one of the key elements of the intrinsic immune system in mature organs, and they primarily function as an immunological defense to keep the internal environment at homeostasis (Wynn and Vannella, 2016; Duan, 2018), and macrophages are resident immune cells in the cochlea (Liu et al., 2018; He et al., 2020). They play a continuous monitoring role in the homeostatic environment, thus helping maintain tissue homeostasis under pathological and physiological conditions (Ito et al., 2022).

Cochlear resident macrophages exhibit remarkable plasticity, allowing them to rapidly switch between active phenotypes and adapt to changing neuronal and environmental conditions. (Hough et al., 2022) In the CNS, microglia protruding as resident macrophages appear near presynaptic neurons, where they remain for about 5 min and then retract (Wake et al., 2009). A recent study (Tremblay et al., 2010) found specific attachments of microglia protrusions to presynaptic and postsynaptic regions in the visual cortex of young mice. In the current study, cochlear resident macrophages were heavily activated and highly migratory 1 week after birth, and they clustered near the inner hair cell ribbon synapses, making frequent but transient contacts with the synapses. The resident macrophages in the cochlea moved back to the region around the ganglion and spiral ligament once the ribbon synapse pruning activity was completed and the ribbon synapses and hearing matured. Additionally, the form changed from amoeboid-like to dendritic. This may be related to the potency of macrophage phagocytosis.

To our best knowledge, this study is the first to verify the role of the CX3CL1/CX3CR1 axis in regulating cochlear resident macrophages in synaptic remodeling and hearing maturation. Our findings are consistent with previous research that CX3CL1 is almost completely produced by neurons (Harry, 2013; Madrigal et al., 2017) and that the CX3CR1 receptor is almost completely expressed on the surface of resident macrophages (Parkhurst et al., 2013). In general, it is thought that the binding of soluble CX3CL1 to its receptor CX3CR1 maintains microglia in a “quiescent” or “off” state (Boche et al., 2013), thus pruning and refining cochlear banded synapses at different stages of auditory development. In the CNS, fractalkine receptor-driven EGFP expression has emerged as a reliable marker for microglial recognition during development (Hsu et al., 2006; Meghraoui-Kheddar et al., 2020).

The synaptic density increases during development in animals lacking this receptor, indicating a deficiency in synaptic pruning. In addition to the chemokine receptor route, the classical activation pathway of complement cascade receptors also contributes to synaptic clearance by microglia (Hong et al., 2016). c1q is thought to be important in the developing nervous system by controlling synaptic pruning (Werneburg et al., 2020). These signals not only control synaptic remodeling in early neurodevelopment, but also play a crucial role in several neurological diseases. Thus, our findings confirm that mice lacking CX3CR1-expressing cochlear macrophages have an increased number of synapses with reduced synaptic pruning during development. CX3CL1, which is locally secreted, may be essential for synapse identification by macrophages and may play a role in macrophage migration to the brain or proliferation during development. The findings demonstrated that CX3CL1 upregulation promoted synaptic pruning and reduced synaptic density and can result in irreversible hearing loss in mice.

## Conclusion

**Figure 7:**
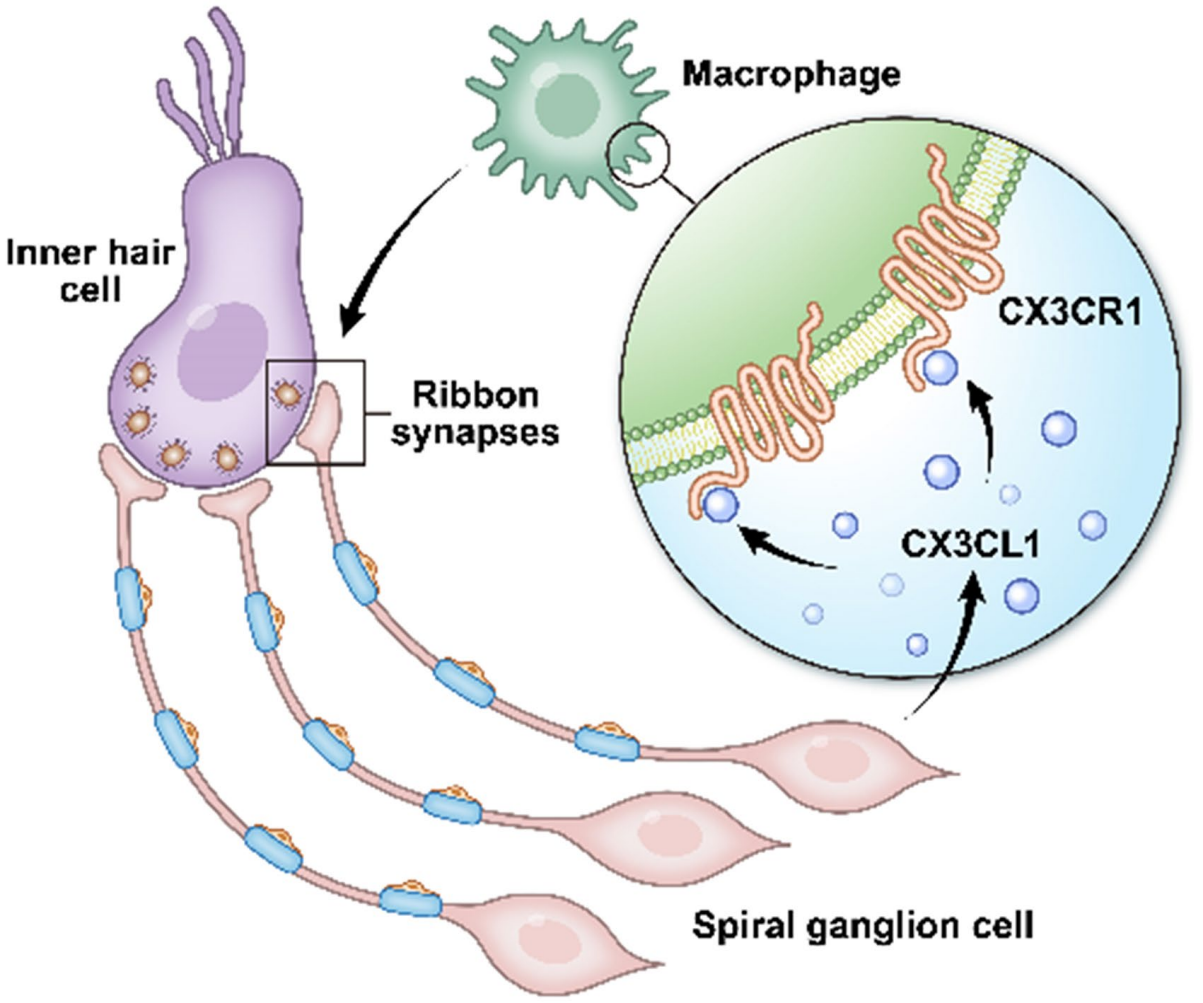
In the cochlea, inner hair cells convert mechanical vibrations into electrical signals *via* ribbon synapses at the base of cells to the auditory nerve, thereby allowing sound information to travel up the auditory pathway to the brain for processing. Cochlear spiral neurons secrete CX3CL1, which binds to CX3CR1, a specific receptor on the surface of macrophages, to phagocytose the excess weak ribbon synapses around inner hair cells during hearing development, participating in ribbon synaptic pruning.

In conclusion, inhibition of CX3CR1 expression on the surface receptors of resident macrophages in the cochlea leads to impaired pruning and refinement of cochlear ribbon synapses, resulting in severe hearing loss, as confirmed in mice in the CX3CR1 inhibition group. Abnormal ABR test results were still observed at P28, providing further evidence that this hearing loss was long-lasting. CX3CL1 neuronal secretion similarly affected cochlear ribbon synapses and hearing function in adult mice. These findings provide evidence that prior to the start of hearing in postnatal mice, macrophages are involved in ribbon synapse remodeling *via* the CX3CL1/CX3CR1 axis, which is necessary for the development of hearing (Figure 7). Collectively, these findings demonstrate that macrophages play a key role in auditory development and maturation. This study suggests that developing ribbon synapses may be potential therapeutic targets for hearing protection in some forms of hearing impairment linked to cochlear macrophages (Bhuskute and Page, 2021; Noulaheu, 2021).

## Data availability statement

The raw data supporting the conclusions of this article will be made available by the authors, without undue reservation.

## Ethics statement

The animal study was reviewed and approved by Animal Ethics Committee of Capital Medical University.

## Author contributions

KL contributed to the design of the study, and analyzed and interpreted the result. SG contributed to the design of the study. XS, YL, RG, QY, SL, QT, Z-RC, and JX conducted the experiments and analyzed the generated data. XS and YL participated in the design and completion of supplementary experiments and wrote the manuscript. All authors contributed to the article and approved the submitted version.

## Funding

This work was supported by the National Natural Science Foundation of China (82071037, 81770997, 81830030, and 82071054); The Joint Funding Project of Beijing Natural Science Foundation and Beijing Education Committee (KZ201810025040).

## Conflict of interest

The authors declare that the research was conducted in the absence of any commercial or financial relationships that could be construed as a potential conflict of interest.

## Publisher’s note

All claims expressed in this article are solely those of the authors and do not necessarily represent those of their affiliated organizations, or those of the publisher, the editors and the reviewers. Any product that may be evaluated in this article, or claim that may be made by its manufacturer, is not guaranteed or endorsed by the publisher.
