# Supplementary figures and images for: Cochlear resident macrophage mediates development of ribbon synapses via CX3CR1/CX3CL1 axis
Source: Front Mol Neurosci. 2022 Nov 28;15:1031278. doi: 10.3389/fnmol.2022.1031278 (PMC9742371; doi:10.3389/fnmol.2022.1031278)

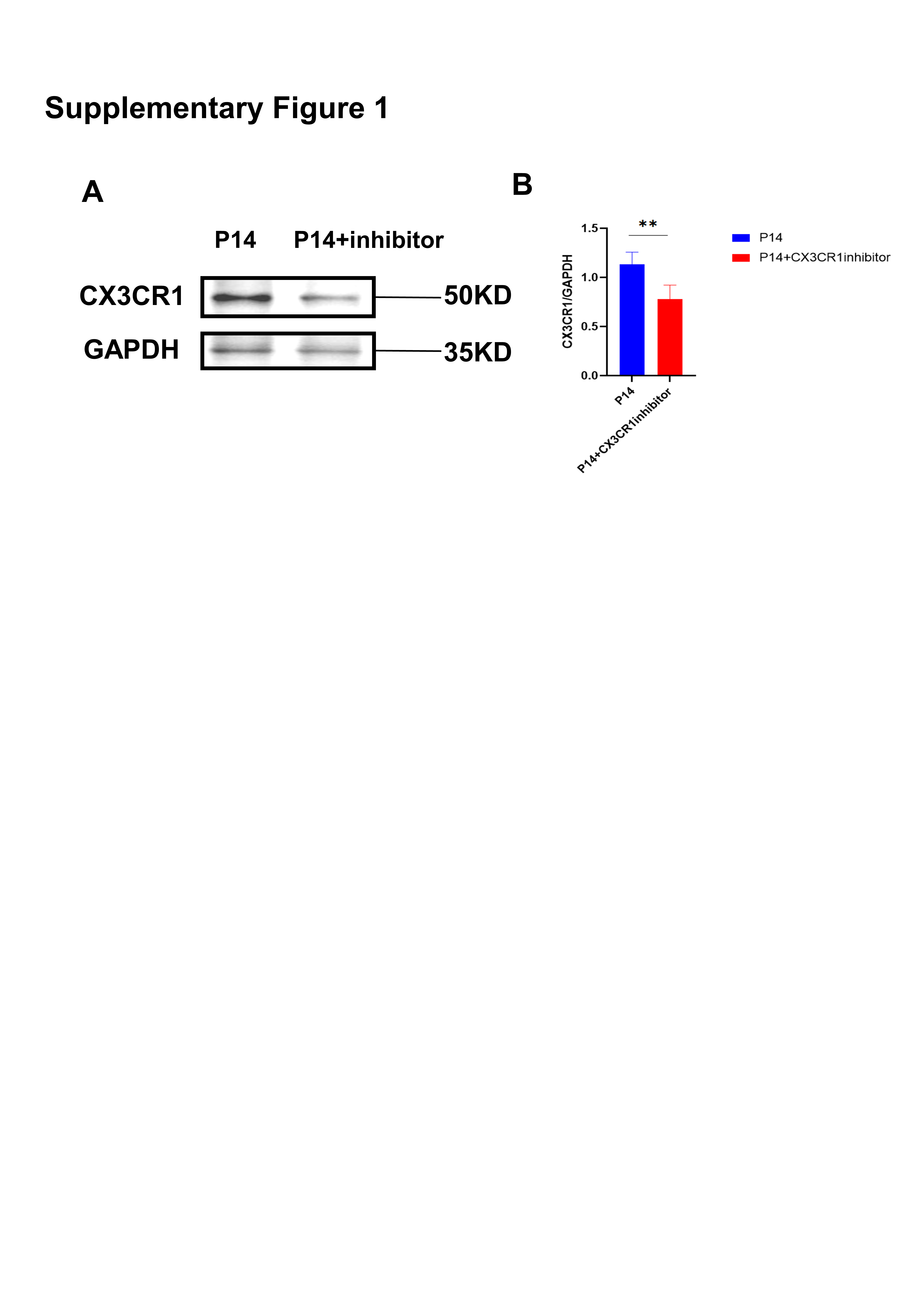

Supplement: Supplementary file 1 [file Image_1.TIF]

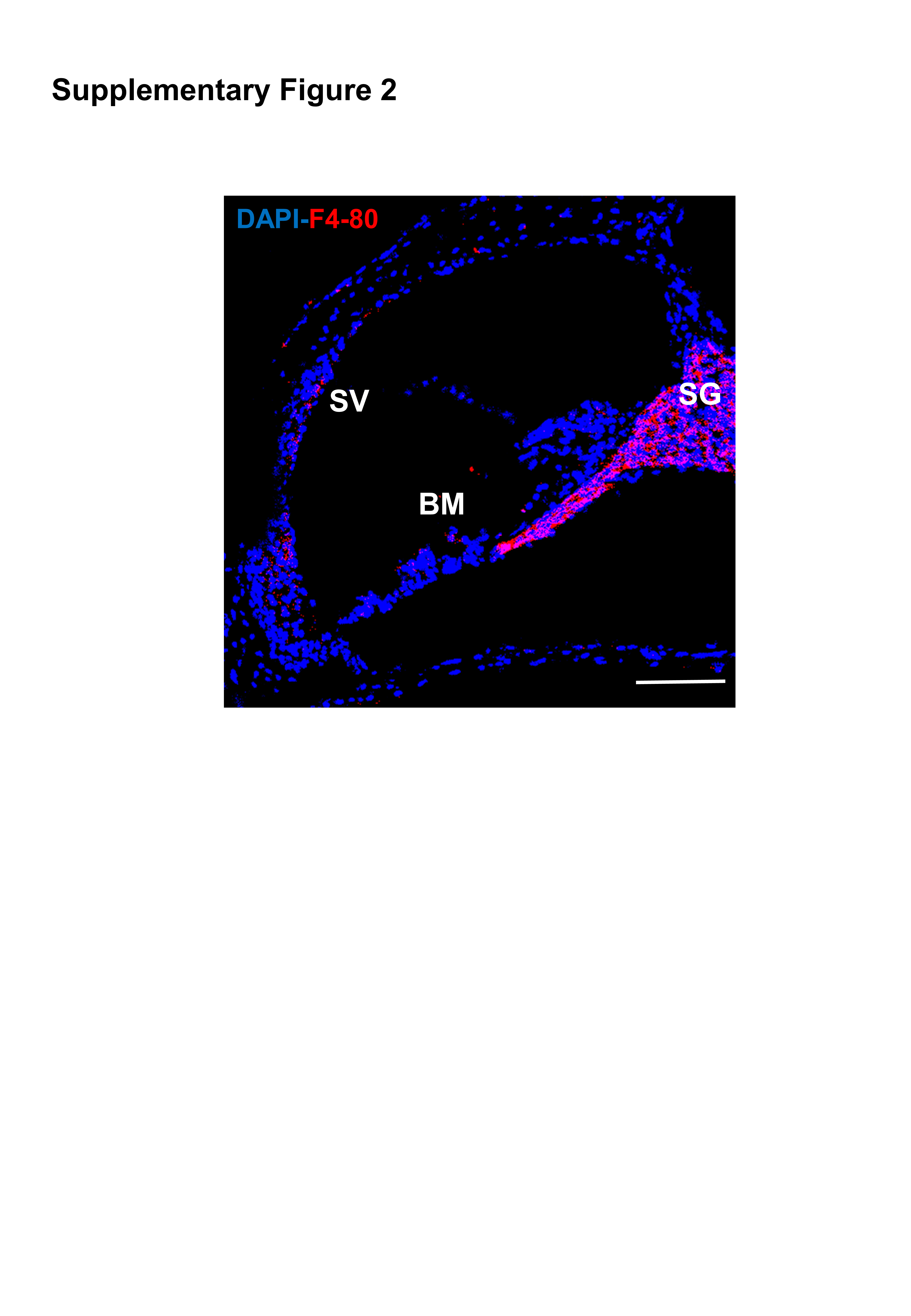

Supplement: Supplementary file 2 [file Image_2.TIF]

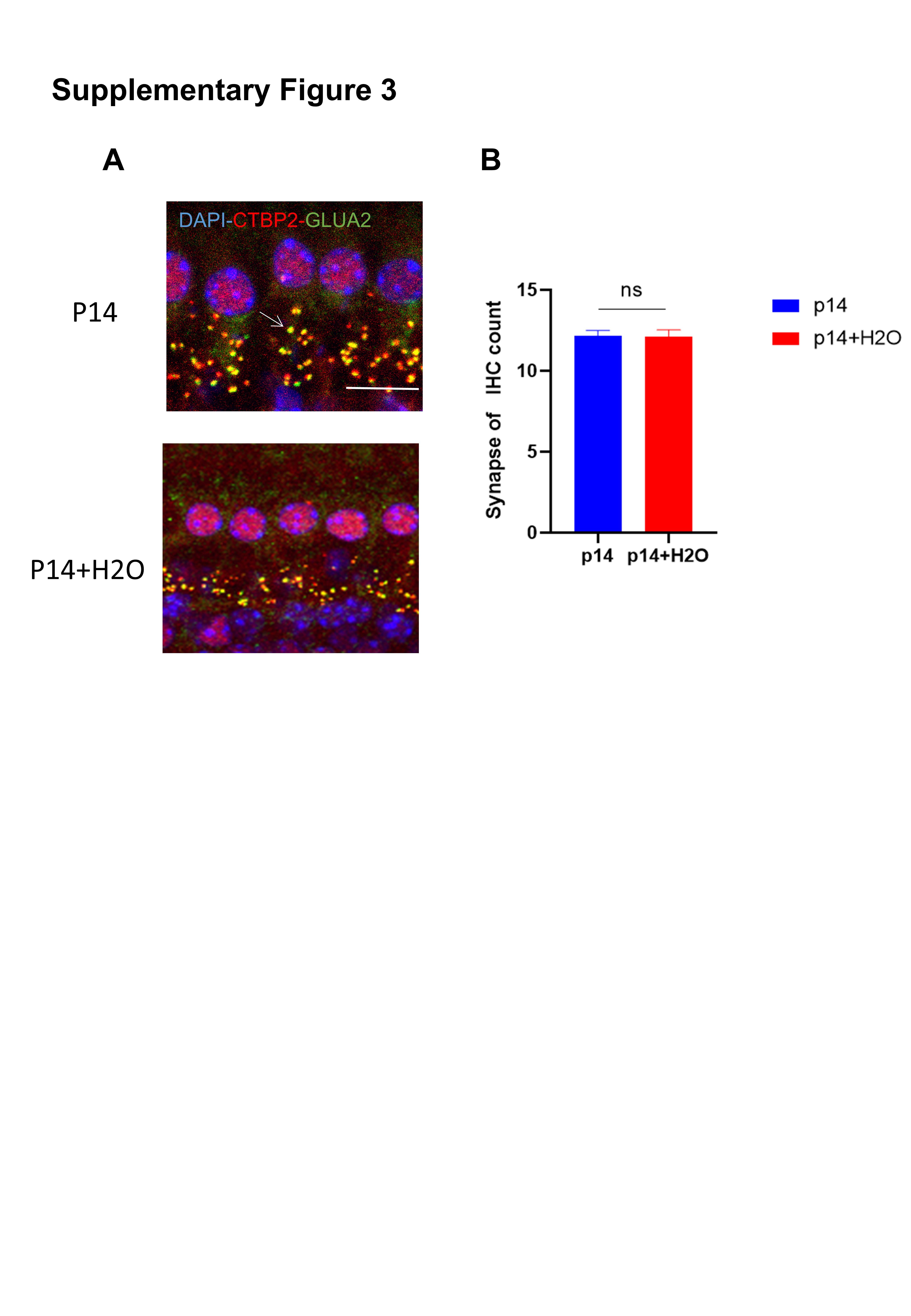

Supplement: Supplementary file 3 [file Image_3.TIF]
